# Supplementary material for: Characterising time-on-task effects on oscillatory and aperiodic EEG components and their co-variation with visual task performance
Source: Imaging Neurosci (Camb). 2025 May 2;3:imag_a_00566. doi: 10.1162/imag_a_00566 (PMC12319923; doi:10.1162/imag_a_00566)
Supplement: Supplementary Material [file imag_a_00566-supp.pdf]

Supplementary Material

Characterising time-on-task effects on oscillatory and aperiodic EEG components and their co-variation with visual task performance.

Martina Kopčanová, Gregor Thut[2], Christopher SY Benwell[1*], Christian Keitel[1*]

@ corresponding author - email: mkopcanova@dundee.ac.uk

* joint senior authors

Affiliations

1 Psychology, University of Dundee, Nethergate, DD1 4HN Dundee, UK **|** **2** Centre de Recherche Cerveau et Cognition (CerCo), CNRS UMR5549 and Université de Toulouse, 31059 Toulouse, France

#	Author	Affiliation	ORCID	Social Media
1	Martina Kopčanová	1	0009-0004-0300-3343	
2	Gregor Thut	2	0000-0003-1313-4262	
3	Christopher SY Benwell	1	0000-0002-4157-4049	
4	Christian Keitel	1	0000-0003-2597-5499	@ckeitelsci.bluesky.social (BlueSky)

Keywords

EEG, neural oscillations, alpha, time on task, reaction time

Table S1 Results of one-way ANOVAs with evidence discriminability as IV and RT as DV tested separately at each block.

**Block**	**F(4,140)**	***p**gg*
1	**24.068**	**<.0001** [a]
2	**12.215**	**<.0001** [b]
3	**16.013**	**<.0001** [c]
4	**6.627**	**.003** [d]
5	**10.905**	**.0001** [e]

The follow up multiple comparisons were significant p < .05 between the following blocks:

[a, b, c] block 1 versus 2, 3, 4, and 5; and block 2 vs 3, 4, and 5.

[d] block 1 vs 2 and 4.

[e] block 1 versus 2, 3, 4, and 5.

Table S2 Results of one-way ANOVAs with block as IV and RT as DV tested separately at each evidence discriminability level.

**Difficulty level**	**F(4,140)**	***p**gg*
**Hard to Easy**		
1	**33.952**	**<.0001** [a]
2	**44.108**	**<.0001** [b]
3	**39.290**	**<.0001** [c]
4	**28.340**	**<.0001** [d]
5	**22.632**	**<.0001** [e]

The follow up multiple comparisons were significant p < .05 between the following blocks:

[a] block 1 versus 2, 3, 4, and 5; and block 2 vs blocks 4

[b] block 1 versus 2, 3, 4, and 5; and block 2 vs blocks 4 and 5

[c, d, e] block 1 versus 2, 3, 4, and 5

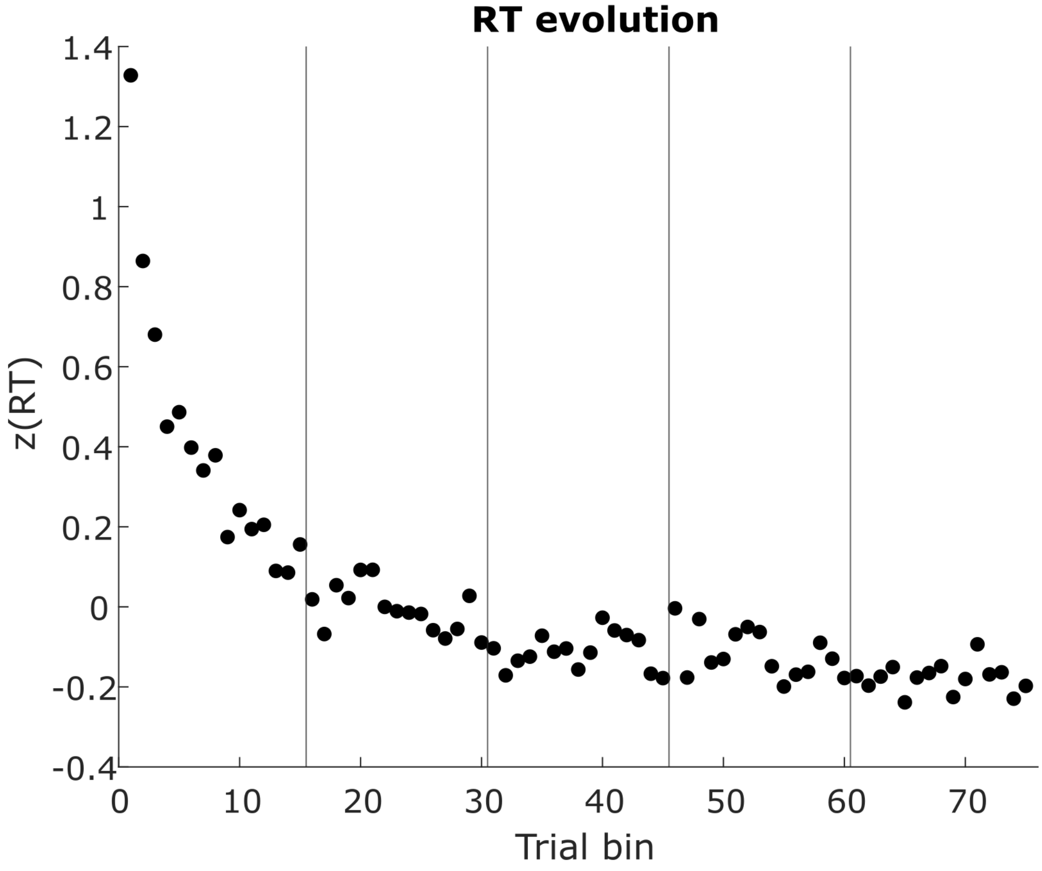

Figure S1: Response reaction times evolution plot. Mean (z-scored) RTs per bin of 10-12 trials in each experimental block were calculated, similar to alpha and beta power in Figure 4 in the main text.

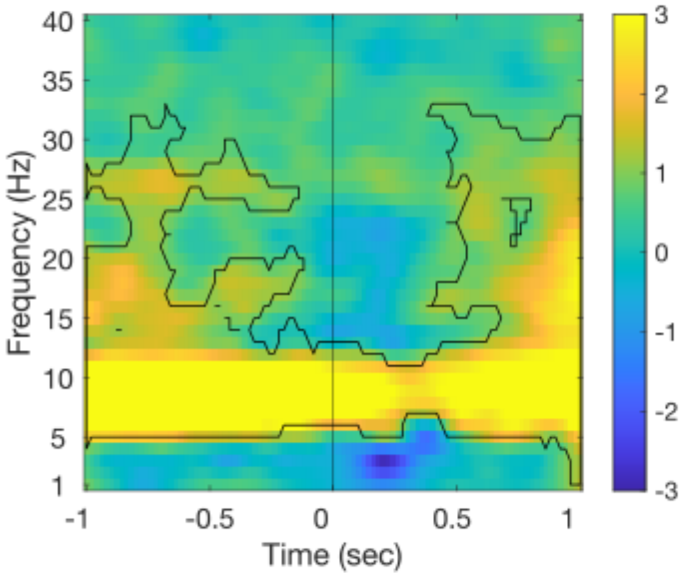

Figure S2: Mean T-values representing the difference in coefficients describing the relationship between RTs and time-frequency power in models with and without time-on-task. Significant clusters (p <.025) are outlined in black. The vertical line denotes stimulus onset.

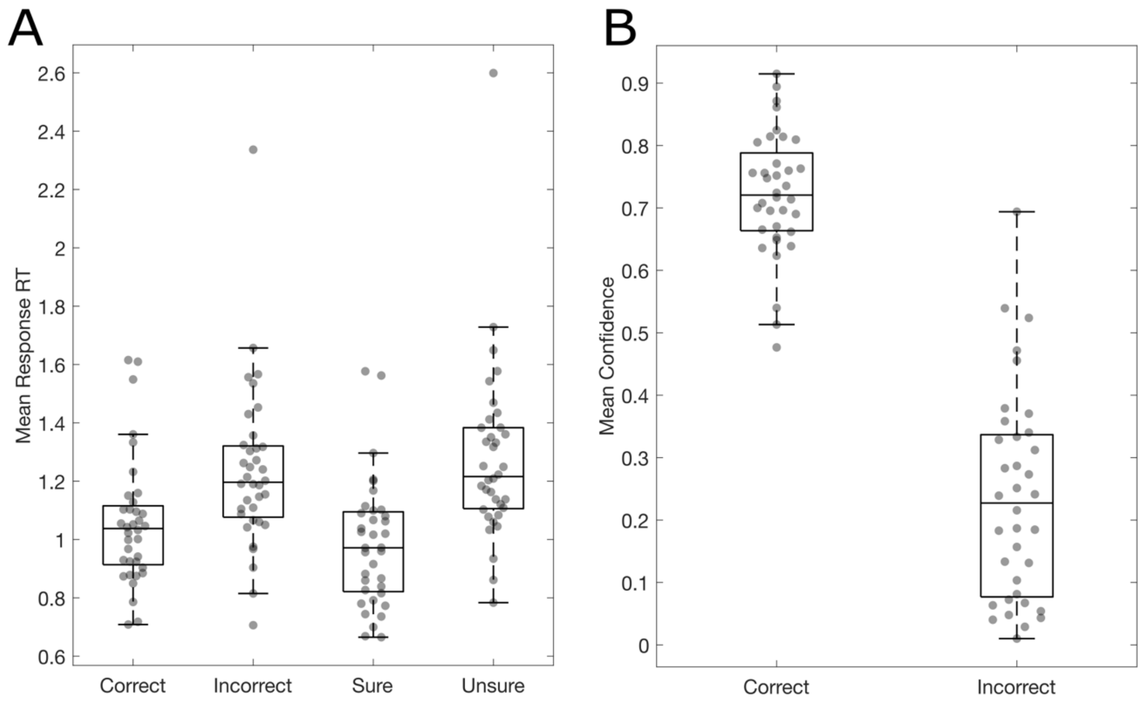

Figure S3: A Mean RTs are plotted for correct/incorrect trials and sure/unsure trials. As expected, RTs were faster on trials where participants responded correctly and were sure in their answer. **B** Mean confidence ratings were higher for correct trials, as expected.
